# Solvent-Focused Gas Chromatographic Determination of Thymol and Carvacrol Using Ultrasound-Assisted Dispersive Liquid–Liquid Microextraction through Solidifying Floating Organic Droplets (USA-DLLME-SFO)

**DOI:** 10.3390/molecules29163931

**Published:** 2024-08-20

**Authors:** Sedigheh Barzegar, Mousab Rehmani, Mahdi Farahmandzadeh, Ghodratollah Absalan, Benson Karimi

**Affiliations:** 1 Professor Massoumi Laboratory, Department of Chemistry, College of Sciences, Shiraz University, Shiraz 71454, Iran; sedighechem62@yahoo.com (S.B.);; 2 Department of Physical and Environmental Sciences, Texas A&M University Corpus Christi, Corpus Christi, TX 78412, USA; 3 Institute of Biotechnology, Shiraz University, Shiraz 71454, Iran; mahdi.farahmandzade@gmail.com

**Keywords:** gas chromatography, thymol, carvacrol, undecanol, solvent focusing, USA-DLLME-SFO, GC-FID

## Abstract

An ultrasound-assisted dispersive liquid–liquid microextraction by solidifying floating organic droplets, coupled to a form of temperature-programmed gas chromatography flame ionization detection, has been developed for the extraction and determination of thymol and carvacrol. This method utilizes undecanol as the extraction solvent, offering advantages such as facilitating phase transfer through solidification and enhancing solvent-focusing efficiency. The optimal gas chromatography conditions include a sample injection volume of 0.2 µL, a split ratio of 1:10, and a flow rate of 0.7 mL min^−1^. The extraction conditions entail an extraction solvent volume of 20 µL, a disperser solvent (acetone) volume of 500 µL, pH 7.0, 7.0% NaCl (3.5 M), a sample volume of 5.0 mL, an ultrasound duration of 10 min, and a centrifuge time of 7.5 min (800 rpm). These conditions enable the achievement of a high and reasonable linear range of 3.5 to 70. 0 μg mL^−1^ for both thymol and carvacrol. The detection limits are found to be 0.95 and 0.89 μg mL^−1^, respectively, for thymol and carvacrol. The obtained relative standard deviations, 2.7% for thymol and 2.6% for carvacrol, demonstrate acceptable precision for the purpose of quantitative analysis.

## 1. Introduction

Thymol and carvacrol constitute the primary bioactive compounds found in the essential oils of numerous plants, including thyme, *Origanum vulgare* (oregano), wild bergamot, and *Thymus vulgaris* [1,2]. These compounds possess antitussive, antibacterial, antifungal, antioxidant, antiparasitic, anticancer, anticarcinogenic, and anti-inflammatory properties [3,4,5,6,7].

Various techniques, including thin-layer chromatography (TLC) combined with densitometry [8], gas chromatography (GC) [4,9,10], high-performance liquid chromatography (HPLC) [11,12,13,14,15,16], and fluorometric detection [17], have been employed to determine thymol and carvacrol in various matrices. Given their volatility at low concentrations, GC is a widely used analytical technique for their determination, with detectors such as a flame ionization detector (FID) [10,18,19,20] and mass spectrometry (MS) [9,10,21,22] commonly utilized.

Prior to analytical chromatography, purification and preconcentration techniques are necessary due to the low concentration of these compounds in complex matrices, representing a bottleneck and time-consuming step in analytical methods [23]. Microextraction methods such as dispersive liquid–liquid microextraction (DLLME) are increasingly employed due to their simplicity, cost-effectiveness, and lower solvent consumption [24,25]. It allows fast extraction kinetics and high enrichment factors compared to classical methods such as liquid–liquid extraction (LLE) [26,27]. DLLME, introduced in 2006 by Assadi et al. [28], involves the use of microliter volumes of the extraction solvent along with a few milliliters of dispersive solvents. The disperser solvent, miscible in both the extraction solvent and the aqueous sample solution, rapidly disperses upon injection into aqueous media, forming a homogenous cloudy phase. This accelerates the extraction process by increasing the contact area between the extraction solvent and the aqueous phase, facilitating the transfer of the target analytes from the aqueous phase to the extraction solvent. The dense extraction solvent obtained after centrifugation settles as the sedimented phase for further analysis [28,29,30,31].

Despite its advantages, DLLME has drawbacks, including the use of toxic halogenated organic solvents that are environmentally unfriendly and possess higher densities than water, making collection challenging. Dispersive liquid–liquid microextraction based on the solidification of floating organic drops (DLLME-SFO), introduced by Leong and Huang in 2008, addresses these drawbacks [32]. In DLLME-SFO, extraction solvents with low melting points and densities lower than water are utilized, allowing extraction solvent droplets to float on the surface post-centrifugation and be easily collected after solidification at low temperatures, without requiring specific holders such as micro syringe tips, hollow fibers, or polychloroprene rubbers [32]. Ultrasonic radiation, a potent aid that accelerates various analytical process steps, has gained acceptance recently due to its ability to enhance the mass transfer rates of analytes from the aqueous phase to the extraction solvent, reduce solvent consumption, and easily integrate with other extraction techniques such as DLLME-SFO [33,34,35,36,37].

The aim of this study is to develop an ultrasound-assisted dispersive liquid–liquid microextraction by the solidifying floating organic droplets (USA-DLLME-SFO) method, coupled to a temperature-programmed GC-FID, utilizing undecanol as the solvent. This approach aims to separate and quantify thymol and carvacrol, leveraging the solvent-focusing capabilities of GC-FID.

## 2. Results and Discussion

### 2.1. Optimization of the Extraction Conditions

Several experimental variables, such as the volume of extraction solvent, type and volume of the dispersion solvent, pH, type of buffer, ionic strength, sample volume (breakthrough volume), centrifugation time, and ultrasonication, were optimized to enhance the efficiency of the USA-DLLME-SFO method. Thymol and carvacrol, both at a concentration of 5.0 mg L^−1^, along with benzene-1, 2-diol (catechol) at a concentration of 1000.0 mg L^−1^ as the internal standard (to correct the variation of the injection volumes), were used. Given that the log P_o/w_ value of catechol is 0.9 [38], it is evident that the extraction efficiency of this internal standard is considerably lower compared to the analytes (with the log P_o/w_ values of thymol = 3.3 and carvacrol = 3.5) [38]. To ensure comparative peaks for the internal standard and the analytes, a final concentration of 1000.0 mg L^−1^ was employed for catechol in the sample. The relative peak area of each analyte (I_signal_) to the internal standard peak area (I_IS_) was used as the analytical signal.

#### 2.1.1. Selection of Extraction Solvent (1-Undecanol) Volume

**Figure 1:**
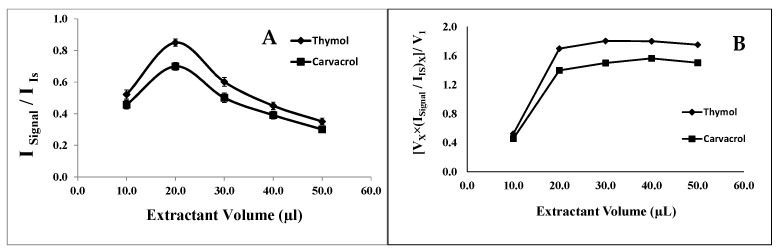
Effect of extraction solvent. (**A**) Effect of the1-undecanol volume on the analytical signal in the determination of thymol (5.0 mg L^−1^) and carvacrol (5.0 mg L^−1^). Experimental conditions: disperser solvent (acetone) = 0.50 mL; (NaOH 1.0 M) pH 7.0; % NaCl = 7.0%; sample volume = 5.0 mL; ultrasound duration time = 10.0 min; centrifugation duration time (800 rpm) = 7.5 min (n = 3). (**B**) Effect of the dilution of the analytes on the optimization of the extraction solvent (1-undecanol) volume in the determination of thymol and carvacrol.

To investigate the effect of the extraction solvent volume on the analyte extraction efficiency, aliquots ranging from 5.0 to 50.0 μL of 1-undecanol were examined while keeping the other experimental conditions constant. The signal intensity of the analytes increased with the increasing volume of 1-undecanol from 10.0 to 20.0 μL and then decreased (see Figure 1A). This phenomenon could be attributed to the dilution of the analytes at a higher extractant volume, resulting in a smaller analytical signal.

To demonstrate this, the analytical signals obtained at all the tested extractant volumes were adjusted by multiplying each signal by the volume of the extractant and dividing the result by the lowest extract volume utilized. The resulting corrected analytical signals were then plotted against the extractant volume, as shown in Figure 1B. As is evident from the figure, the corrected signal stabilizes at volumes of 20.0 μL and higher, indicating that the decrease in the signal intensity observed in Figure 1A at higher extractant volumes is indeed due to the analyte dilution. Consequently, 20.0 μL of 1-undecanol was selected as the optimal extraction volume for subsequent experiments. It is worth noting that micro drops could not practically form on the surface of the aqueous sample when using 1-undecanol volumes less than or equal to 5.0 μL. Typically, the volume of the extraction solvent should be sufficient to extract as many analytes as possible while ensuring an adequate volume of extracted phase for further chromatographic analysis. Hence, extractant volumes less than 10.0 μL have not been practically feasible in the developed system.

#### 2.1.2. Selection of Disperser Solvent

**Figure 2:**
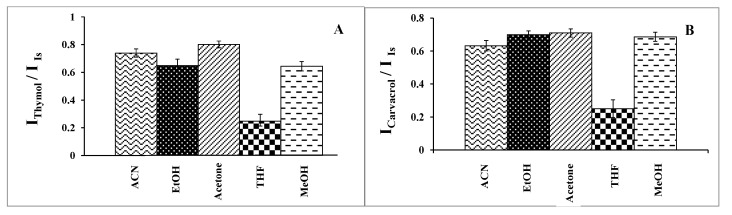
Effect of the type of disperser solvent on the analytical signal under experimental conditions: extractant solvent (1-undecanol) = 20.0 µL; disperser volume = 0.5 mL; pH 7.0; % NaCl = 7.0%; sample volume = 5.0 mL; ultrasound duration time = 10.0 min; and centrifuging duration time (at 800 rpm) = 7.5 min. (n = 3). (**A**) Determination of thymol (5.0 mg L^−1^). (**B**) Determination of carvacrol (5.0 mg L^−1^).

The selected disperser solvent must meet several criteria. It is essential to consider not only its miscibility with both the extraction solvent and the aqueous sample but also its toxicity and cost. It should be noted that the disperser solvent aids in producing very fine, dispersed droplets of the extraction solvent throughout the aqueous sample solution, thereby significantly increasing its contact area with the aqueous sample solution. In this work, ACN, MeOH, EtOH, THF, and acetone were tested. Extraction of the analytes was carried out using 0.5 mL of each solvent with 20.0 μL of 1-undecanol under the operating conditions of pH 7.0, % NaCl = 7.0%, a sample volume of 5.0 mL, an ultrasound duration time of 10.0 min, and a centrifuge duration time (at 800 rpm) of 7.5 min. According to the results (Figure 2), acetone was selected as the disperser solvent.

As is evident from Table 1, THF has a positive log P_o/w_, indicating low solubility in aqueous media. ACN, MeOH, and EtOH possess negative log P_o/w_ values, suggesting low abilities to dissolve organic extractants. It appears that acetone has a near zero log P_o/w_ value, indicating not only the adequate solubility of 1-undecanol in acetone but also sufficient solubility in water. On the other hand, acetone is more polar than the other potential dispersers, making it easily solvated in water to disperse the organic extraction solvent.

#### 2.1.3. Selection of Disperser Solvent Volume

The disperser solvent must significantly increase the interfacial area between the extraction solvent and the aqueous sample solution to enhance the extraction rate. Therefore, the volume of the disperser solvent (acetone in this study) needs to be optimized. It should be noted that at low volumes of the disperser solvent, there is insufficient formation of fine droplets of extraction solvent to create a well-clouded state, resulting in reduced extraction efficiency. Conversely, at high volumes of the disperser solvent, its higher solubility in the aqueous phase compared to the organic phase often reduces the polarity of the aqueous phase, leading to decreased analyte extraction.

**Figure 3:**
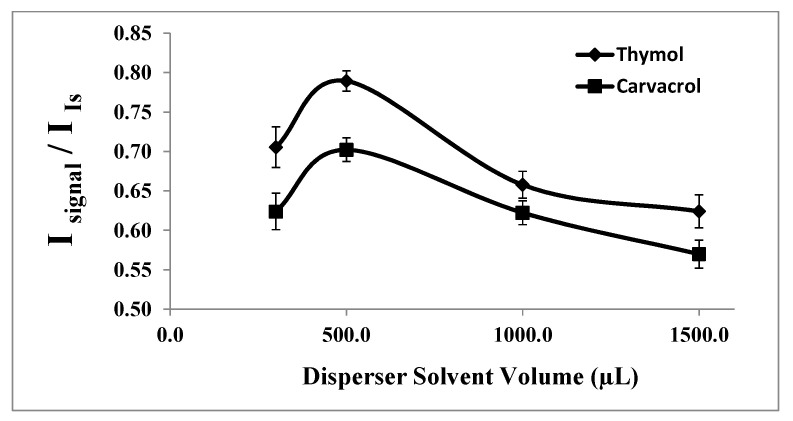
Effect of the disperser solvent (acetone) volume on the analytical signal in the determination of thymol (5.0 mg L^−1^) and carvacrol (5.0 mg L^−1^). Experimental conditions: extractant solvent (1-undecanol) = 20.0 µL; pH 7.0; % NaCl = 7.0%; sample volume = 5.0 mL; ultrasound duration time = 10.0 min; and centrifuging duration time (at 800 rpm) = 7.5 min (n = 3).

To investigate the effect of the disperser solvent volume on the extraction of thymol and carvacrol, various volumes of acetone (0.30, 0.50, 1.00, and 1.50 mL) with 20.0 μL of 1-undecanol were tested under constant experimental conditions. Upon careful examination of the results, as shown in Figure 3, 0.50 mL of acetone was determined to be the optimal volume for further studies.

#### 2.1.4. Optimization of pH of Aqueous Sample Solution

Considering the pKa values of thymol (pKa = 10.62) and carvacrol (pKa = 10.42), it is evident that they do not ionize in acidic and neutral solutions, suggesting that the extraction efficiencies might not be pH-dependent in those media.

Compounds such as alcohols and phenols (thymol and carvacrol), which contain an -OH group attached to a hydrocarbon, are very weak acids. Alcohols are typically so weakly acidic that, for normal lab purposes, their acidity is often disregarded. However, phenol exhibits sufficiently acidic properties to be recognizable, albeit still functioning as a very weak acid [39]. The hydrogen ion within the phenol structure can transfer to a base, resulting in the formation of a stable phenoxide ion due to the delocalization of negative charge around the ring on the oxygen atom. Consequently, the ion formed becomes more soluble in water and less likely to be extracted into an organic solvent. This implies that the pH of the medium impacts the extraction efficiency of thymol and carvacrol [39].

**Figure 4:**
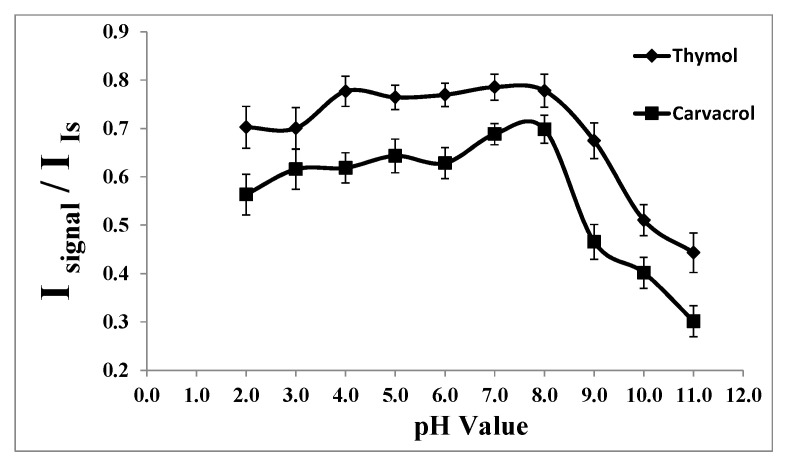
Effect of the pH on the analytical signal in the determination of thymol (5.0 mg L^−1^) and carvacrol (5.0 mg L^−1^). Experimental conditions: extractant solvent (1-undecanol) = 20.0 µL; disperser solvent (acetone) = 0.50 mL; % NaCl = 7.0%; sample volume = 5.0 mL; ultrasound duration time = 10.0 min; and centrifuging duration time (at 800 rpm) = 7.5 min (n = 3).

In our investigation, we examined the extraction of thymol and carvacrol across a pH range of 2.0 to 12.0 using 1.0 mol L^−1^ HCl and 1.0 mol L^−1^ NaOH solutions. As depicted in Figure 4, the signal remains stable within the pH range of 2.0 to 8.0. However, at higher pH values, there is a reduction in the signal, likely attributed to the ionization of the analytes and the formation of sodium phenoxide, leading to their increased solubility in aqueous media and consequently reduced extraction into the extracting solvent. Therefore, pH 7.0 was identified as the optimal pH for subsequent studies.

#### 2.1.5. Type of Solution for Adjusting the pH

**Figure 5:**
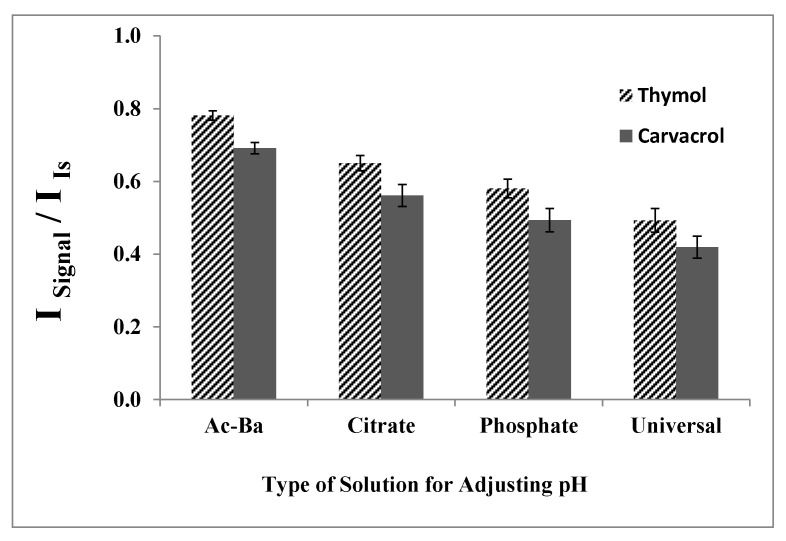
Effect of the type of buffer solution on the analytical signal in the determination of thymol (5.0 mg L^−1^) and carvacrol (5.0 mg L^−1^). Experimental conditions: extractant solvent (1-undecanol) = 20.0 µL; disperser solvent (acetone) = 0.50 mL; pH 7.0; % NaCl = 7.0%; sample volume = 5.0 mL; ultrasound duration time = 10.0 min; and centrifuging duration time (at 800 rpm) = 7.5 min.

Various buffer solutions at pH 7.0 were employed under constant experimental conditions to determine the most suitable buffer. Phosphate, citrate, and universal buffers, as well as hydrochloric acid or sodium hydroxide, were utilized for the pH adjustment. As depicted in Figure 5, utilizing HCl along with NaOH to adjust the pH to 7.0 results in higher analytical signals. Consequently, NaOH was selected for the pH adjustment in the subsequent experiments.

#### 2.1.6. Effect of Salt Addition

The addition of salt to an aqueous sample solution can sometimes enhance the extraction efficiency of analytes into the organic phase. However, higher concentrations of salt may reduce the diffusion rates of analytes into the organic phase due to the increased solution viscosity [40]. Therefore, the salt amount must be optimized in DLLME-SFO.

**Figure 6:**
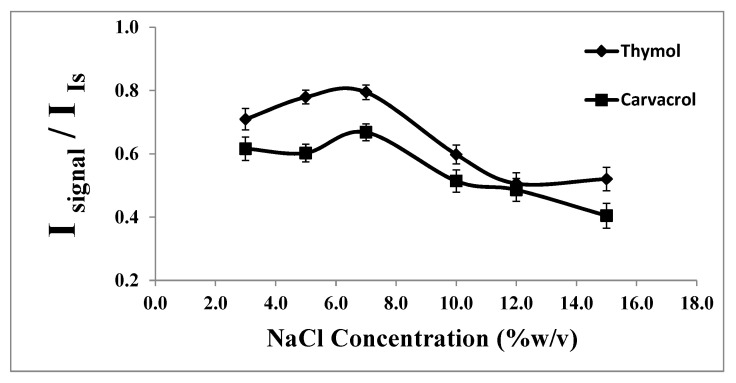
Effect of the % NaCl on the analytical signal in the determination of thymol (5.0 mg L^−1^) and carvacrol (5.0 mg L^−1^). Experimental conditions: extractant solvent (1-undecanol) = 20.0 µL; disperser solvent (acetone) = 0.50 mL; pH 7.0; sample volume = 5.0 mL; ultrasound duration time = 10.0 min; and centrifuging duration time (at 800 rpm) = 7.5 min (n = 3).

The effect of salt addition on the extraction of thymol and carvacrol was evaluated by adding sodium chloride (0.0–15.0%, *w/v*) into the water sample. As illustrated in Figure 6, increasing the NaCl concentration from 0.0 to 7.0% (*w/v*) resulted in an enhanced signal intensity and consequently increased analyte extraction. One possible explanation for this observation is that water molecules form hydration spheres around the salt ions, reducing the amount of water available to dissolve analyte molecules. This reduction in the water availability decreases the solubility of thymol and carvacrol in the aqueous phase, thereby driving more analytes into the extraction solvent [40].

However, the results indicated that with further increases in the NaCl concentration, the analytical signals gradually decreased due to the increased aqueous phase viscosity and reduced diffusion rate of the analytes decreasing during the contact time. Based on these findings, a concentration of 7.0% (*w/v*) of NaCl was chosen as the optimum concentration.

#### 2.1.7. Breakthrough Volume

For the preconcentration of trace analytes, achieving a high preconcentration factor necessitates determining the breakthrough volume of the sample solution. The effect of the sample volume on the microextraction procedure was studied to ascertain the minimum volume that can be effectively utilized [41].

**Figure 7:**
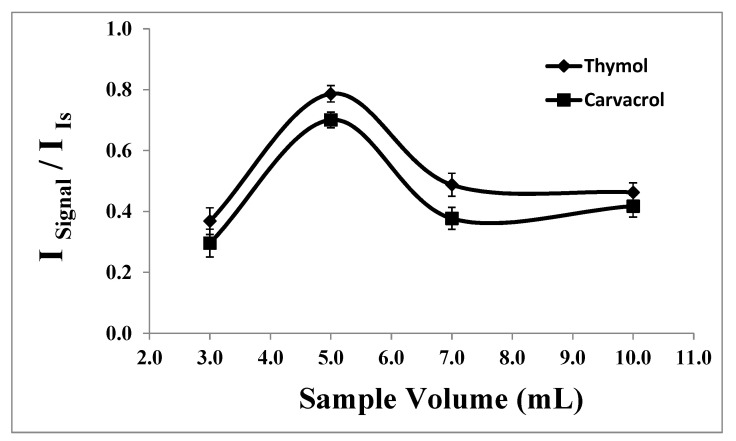
Effect of the sample volume on the analytical signal of thymol (5.0 mg L^−1^) and carvacrol (5.0 mg L^−1^). Experimental conditions: extractant solvent (1-undecanol) = 20.0 µL; disperser solvent (acetone) = 0.50 mL; pH 7.0; %NaCl = 7.0%; ultrasound duration time = 10.0 min; and centrifuging duration time (at 800 rpm) = 7.5 min (n = 3).

Various volumes of sample solutions, each containing 1.66 × 10^−7^ moles of analyte, were prepared in individual glass tubes, ranging from 3.0 to 10.0 mL, under constant experimental conditions. As illustrated in Figure 7, the highest analytical response was attained at a sample volume of 5.0 mL. In smaller sample volumes, the high concentration of the salt inhibits effective interaction between the analytes and the extraction solvent. Conversely, in larger sample volumes, incomplete dispersion of the extraction solvent leads to decreased extraction efficiencies and analytical signals.

The preconcentration factor is calculated as the ratio of the highest sample volume for the analyte (5.0 mL) to the lowest extraction solvent volume (20.0 µL) [41]. In this study, the preconcentration factor was determined to be 250.

#### 2.1.8. Effect of Ultrasound and Centrifugation Period

Ultrasound was employed as a disperser in the ultrasound-assisted microextraction of thymol and carvacrol due to its capability to provide sufficient mechanical and thermal energy, enabling the extraction of these heat-sensitive essential oils at low temperatures. Generally, ultrasound enhances the mass transfer rates between two immiscible phases and facilitates emulsification, thereby improving the efficiency of simultaneous liquid–liquid microextraction analytes. Additionally, ultrasound offers an inexpensive and environmentally friendly method [40].

The analytical signals were examined by varying the ultrasound durations between 6.0, 8.0, 10.0, and 12.0 min, revealing that durations of 10.0 min or longer resulted in higher signals. Similarly, centrifugation times of 2.5, 5.0, 7.5, and 10.0 min at 800 rpm were tested, revealing that durations of 7.5 min or more resulted in enhanced analytical signals due to the more efficient separation of the organic and aqueous phases.

Consequently, an ultrasound duration of 10.0 min and a centrifugation time of 7.5 min were selected for further studies.

### 2.2. Analytical Figures of Merit

**Figure 8:**
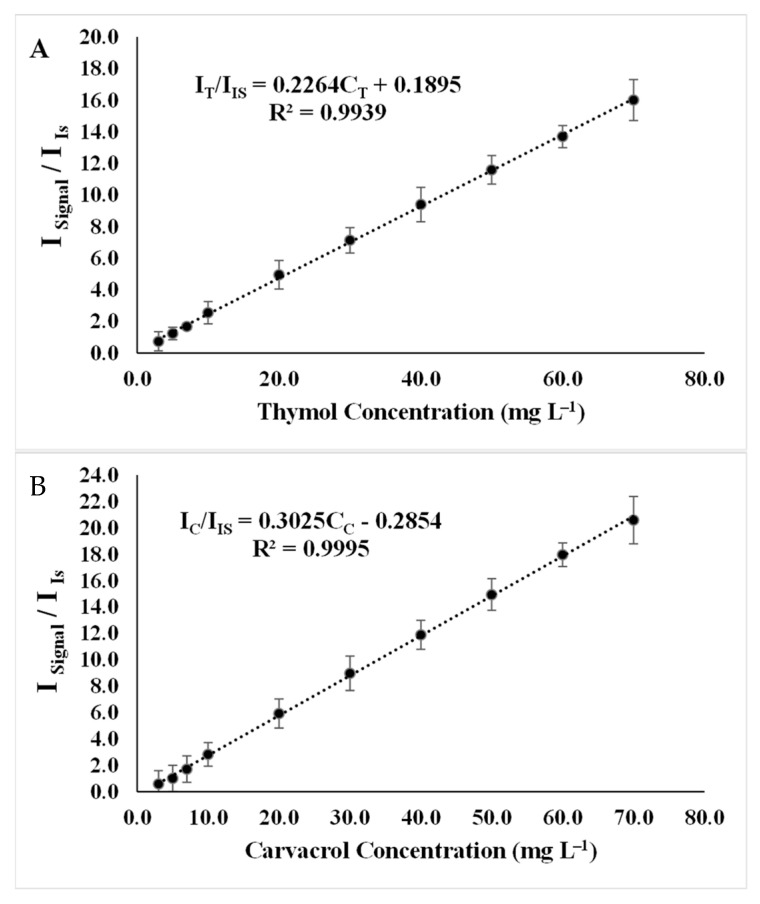
Calibration curve under experimental conditions: extractant solvent (1-undecanol) = 20.0 µL; disperser solvent (acetone) = 0.50 mL; pH 7.0; %NaCl = 7.0%; ultrasound duration time = 10.0 min; and centrifuging duration time = 7.5 min. (I/I_IS_ = relative signal intensity of analyte to signal intensity of internal standard and C_T_ = thymol concentration, C_C_ = carvacrol concentration). (**A**) Thymol. (**B**) Carvacrol.

After optimizing all the experimental parameters, calibration curves were plotted on three different days for 10 concentration levels, with each concentration level replicated three times. The data obtained from these experiments were used for validation studies. It was observed that there exists a strong linear relationship between the relative signal intensity and the concentration of analytes in the concentration range of 3.5 to 70.0 μg mL^−1^ for thymol (Figure 8A) and carvacrol (Figure 8B).

The limit of detection (LOD) was calculated to be 0.95 and 0.89 μg mL^−1^, while the limit of quantification (LOQ) was determined to be 3.16 and 2.96 μg mL^−1^ for thymol and carvacrol, respectively.

**Figure 9:**
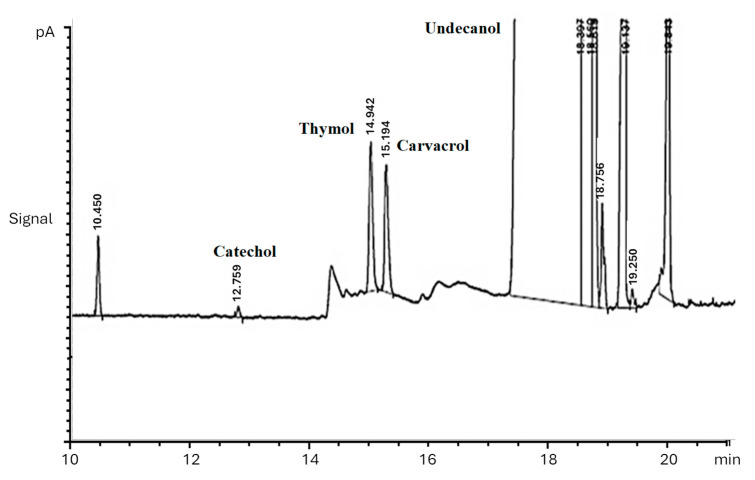
Chromatograph for the determination of thymol and carvacrol. Conditions: extractant solvent: 1-undecanol (20.0 µL); disperser solvent = acetone (0.50 mL); pH 7.0; %NaCl = 7.0%; ultrasound duration time = 10.0 min; centrifuging duration time = 7.5 min.

The precision of the method was assessed through five replicated analyses. The relative standard deviations (RSDs) for thymol and carvacrol are presented in Table 2. Five replicated measurements were conducted to determine both the within-day (RSD less than 3.4) and between-day (RSD less than 5.4) precisions, based on the average of the repeated measurements each day. The average RSD for the within-day determination of thymol was 2.7, and for carvacrol, it was 2.6, as presented in Table 2. A typical chromatogram under optimum conditions is presented in Figure 9.

### 2.3. Enrichment Factor and Extraction Recovery

The enrichment factor (*EF*) is defined as the ratio of the analyte concentration in the extraction phase (*C_O_*) to the initial concentration of the analyte in the source phase, typically an aqueous sample (*C_W_*) [40]:

(1) $$EF=\frac{C_{O}}{C_{W}}$$

The extraction recovery (*ER*) is defined as the percentage of the amount of analyte extracted in the organic phase (*V_O_* mL) from the aqueous sample (*V_W_* mL), and it is calculated according to the following equation [32]:

(2) $$ER\%=\frac{C_{O}\times V_{O}\;}{C_{w}\times V_{w}}\times 100$$

The calculated *EF* of 138.1 for thymol and 136.6 for carvacrol, along with the *ER*% of 55.2 for thymol and 54.6 for carvacrol, are presented in Table 3.

### 2.4. Robustness

Robustness evaluates the ability of an analytical method to withstand the small changes (within 5%) in practical effective parameters that may vary (directional changes) during operation [42]. In fact, these small variations in the effective parameters can impact the measurement results and should be assessed during method validation. The reliability of the analysis can be evaluated through robustness testing, which measures the method’s ability to remain unaffected by minor changes to the effective method parameters.

To evaluate the robustness, small variations in the effective parameters were introduced, including the pH, %NaCl, extraction and disperser solvent volumes given in Table 4, and the resulting quantitative influences were determined. The results, as shown in Table 4, indicated that the presence of negligible variation in the analytical signal, despite the instability of the effective parameters, demonstrates the method’s robustness.

### 2.5. Analytical Approaches for Determination of Thymol and Carvacrol

Compared to other methods, the presented technique is notable for its speed, simplicity, low cost, and efficiency, as it consumes minimal solvents and ensures the essential oil extraction safety.

The use of low-density and low-melting-point organic solvents, such as 1-undecanol, as the extraction solvent enables the easy collection of the extraction micro droplets by solidification, thereby facilitating phase transfer. Additionally, the use of a low-toxicity extraction solvent makes this technique environmentally friendly. The USA-DLLME-SFO method exhibits excellent clean-up capabilities and effectively eliminates matrix effects. It demonstrates good precision, selectivity, stability, and robustness, making it suitable for routine monitoring of thymol and carvacrol.

The comparison of these findings with those of previous works listed in Table 5 highlights the environmental friendliness and acceptable analytical figures of merit of the developed technique for the determination of these oils.

## 3. Materials and Methods

### 3.1. Reagents

The thymol and carvacrol standards, catechol (1, 2-dihydroxy benzene), sodium chloride, acetone, tetrahydrofuran (THF), methanol (MeOH), ethanol (EtOH), acetonitrile (ACN), citric acid, phosphoric acid, hydrochloric acid, boric acid and 1-undecanol (>99%, *w/w*) were purchased from Merck Chemicals Company (Darmstadt, Germany). The sodium hydroxide was bought from KANTO Chemical Company (Tokyo, Japan), and the acetic acid was obtained from Panreac Quimica SA (Barcelona, Spain). All the experiments were performed using deionized water. The thymol and carvacrol stock solutions were individually prepared by dissolving 5.0 mg thymol, 5.2 µL carvacrol in ethanol in a 5.0 mL volumetric flask, and 75.0 mg catechol (as internal standard) in water in a 100.0 mL volumetric flask, and they were stored in a refrigerator. Different binary standard solutions were prepared from these stock solutions. A carefully measured quantity of the internal standard substance was introduced into each standard and sample solution.

### 3.2. Apparatus

The GC analysis was performed by using an Agilent gas chromatograph model 7890A (Santa Clara, CA, USA) equipped with an HP-1 methyl siloxane column (30 m in length × 250 µm × 0.25 μm) equipped with the FID. A digital ultrasound cleaner, trademark: Codyson (Shenzhen, China), model: CD-4820 was used.

### 3.3. GC Analysis Condition

Nitrogen was used as the carrier gas at a constant flow rate of 0.7 mL min^−1^. The oven temperature was increased from 45 to 120 °C at a rate of 6 °C min^−1^ and held for 5 min, then raised to 260 °C at a rate of 50 °C min^−1^ and then held for 5 min. The temperatures of the injection and detection systems (FID) were 240 and 280 °C, respectively. A sample volume of 0.2 µL was injected and the split ratio was 1:10.

### 3.4. Sample Preparation

Each sample was prepared by pouring 2.21 mL of distilled water, 20.0 µL of thymol in ethanol solution, 20.0 µL of carvacrol in ethanol solution, 1.0 mL of catechol in ethanol solution, and 1.75 mL of NaCl (final concentration 7.0% *w/v*) into a sample tube. The sample was then neutralized to pH 7.0 by adding 1.0 M NaOH. A mixture of 0.50 mL acetone and 20.0 µL 1-undecanol was dispersed in the solution using a syringe. Each sample was subsequently placed in an ultrasound bath for 10.0 min and then centrifuged for 7.5 min (800 rpm). The sample tube was refrigerated because undecanol has a melting point of 11 to 14 °C; thus, it could solidify rapidly compared to water and separate from the aqueous phase. The organic phase was now ready to be injected into the GC.

## 4. Conclusions

USA-DLLME-SFO and GC-FID were coupled for the extraction and determination of essential oils, marking the first time such a method has been employed. Ultrasonic-assisted extraction was leveraged to effectively extract the essential oils under moderate conditions (room temperature and atmospheric pressure) in a brief period.

The combination of the technique with GC-FID has proven to be an efficient and economical approach for the analysis of thymol and carvacrol. Moreover, the main advantage of the developed method lies in its simplicity and low cost, rendering it suitable for routine applications in biomedical analysis laboratories.

## Figures and Tables

**Table 1:** Properties of some solvents as a possible disperser in USA-DLLME-SFO [38].

Disperser Solvent	Molar Mass (g mol^−1^)	Miscibility in Water	Dipole Moment (Debye)	Log P_o/w_
Acetonitrile	41.05	Miscible	3.92	−0.334
Methanol	32.04	Miscible	1.69	−0.69
Ethanol	46.07	Miscible	1.69	−0.18
Tetrahydrofuran	72.11	Miscible	1.63	+0.35
Acetone	58.08	Miscible	2.91	−0.042

**Table 2:** Within-day and between-day precisions (n = 5) obtained for different concentrations of thymol and carvacrol.

Precision	Analyte	Concentration (mg L^−1^)	RSD%
Within-day	Thymol	5.0	3.2
		10.0	2.3
		50.0	2.5
	Carvacrol	5.0	3.4
		10.0	2.7
		50.0	1.8
Between-day	Thymol	5.0	5.1
		10.0	4.2
		50.0	1.8
	Carvacrol	5.0	5.4
		10.0	2.6
		50.0	2.1

**Table 3:** Enrichment factor (EF) and extraction recovery (ER%).

Analyte	*C_W_* ^a^ (mg L^−1^)	*V_W_* ^b^ (µL)	*C_O_* ^c^ (mg L^−1^)	*V_O_* ^d^ (µL)	*EF*	*ER*%
Thymol	5	5000	690.4	20	138.1	5.2
Carvacrol	5	5000	683.1	20	136.6	54.6

^a^ Initial concentration of analyte in the aqueous phase. ^b^ Aqueous phase volume. ^c^ Analyte concentration in organic solvent (extraction phase). ^d^ Organic solvent volume.

**Table 4:** Evaluating the robustness of the method at three different experimental condition levels for two different concentrations of thymol and carvacrol.

Experimental Conditions	Analyte	Picked (mg L^−1^)	Found ± SD (mg L^−1^)	Accuracy (RE) ^a^	Recovery% (n = 3)
A	Thymol	5.0 50.0 5.0 50.0	4.8 ± 0.14 49.3 ± 0.32 4.7 ± 0.05 50.5 ± 0.51	−0.040 −0.014 −0.060 +0.010	96.0 98.6 94.0 101.0
	Carvacrol				
B	Thymol	5.0 50.0 5.0 50.0	4.5 ± 0.13 50.4 ± 0.42 4.3 ± 0.81 48.8 ± 0.22	−0.10 +0.032 −0.140 −0.024	90.0 100.8 86.0 97.6
	Carvacrol				
C	Thymol	5.0 50.0 5.0 50.0	5.1 ± 0.16 49.2 ± 0.22 4.6 ± 0.34 49.0 ± 0.47	+0.060 −0.016 −0.080 −0.020	102.0 98.4 92.0 98.0
	Carvacrol				

A. pH 7.0, %NaCl: 7.0, extraction solvent: 20.0 µL, disperser solvent: 0.50 mL. B. pH 7.3, %NaCl: 7.3, extraction solvent: 21.0 µL, disperser solvent: 0.52 mL. C. pH 6.7, %NaCl: 6.7, extraction solvent: 19.0 µL, disperser solvent: 0.48 mL. ^a^ $Relative\;error(RE)=\frac{absolute\;error}{value\;of\;thing\;measured}.$

**Table 5:** Characteristics of some methods reported for the determination of thymol and carvacrol in the literature to be compared with the developed technique.

Method	Solvent	Analyte	LOD (mg mL^−1^)	R^2^	Linear Range (mg mL^−1^)	t_R_^g^ (min)	RSD ^h^ %	Recovery %	Ref
HD-HSME-GC-FID ^a^	Tetradecane, Pentadecane, Hexadecane, Heptadecane	Thymol	1.9 × 10^−3^	0.9944	6.2 × 10^−3^–8.1 × 10^−2^	<20	6.4	89–101	[4]
		Carvacrol	2.3 × 10^−4^	0.9979	1.2 × 10^−3^–8.8 × 10^−2^	<20	11.4	95–116	
UAME-NMSPD-HPLC ^b^	Acetonitrile Methanol	Thymol	2.3 × 10^−7^	0.9995	0.5 × 10^−5^–2.0 × 10^−3^	-	2.1–4.8	95–99	[16]
		Carvacrol	2.1 × 10^−7^	0.9993	0.5 × 10^−5^–2.0 × 10^−3^	-	2.7–4.9	94–98	
HS-SPME-GC-MS ^c^	Methanol	Thymol	8.9 × 10^−7^	0.9994	2.0 × 10^−6^–4.0 × 10^−4^	<7	2.2–11.3	-	[42]
		Carvacrol	5.7 × 10^−7^	0.9997	2.0 × 10^−6^–4.0 × 10^−4^	<7	0.8–9.8	-	
VASE-DLLME- HPLC ^d^	Chloroform Acetonitrile	Thymol	1.6 × 10^−6^	0.9998	0.5 × 10^−5^–4.0 × 10^−3^	<14	1.0–4.8	97	[11]
		Carvacrol	1.6 × 10^−6^	0.9998	0.5 × 10^−5^–4.0 × 10^−3^	<14	1.0–4.8	97	
MMIP-DSPME- HPLC ^e^	Acetonitrile	Thymol	(0.4–1.0) × 10^−5^	0.999	0.4 × 10^−6^–5.0 × 10^−2^	<7	1.0–4.9	97–104	[15]
		Carvacrol	(0.4–1.0) × 10^−5^	0.999	0.4 × 10^−6^–5.0 × 10^−2^	<7	1.7–6.3	97–105	
DLLME-SFO-GC-FID ^f^	Undecanol	Thymol	9.5 × 10^−4^	0.9939	(0.3–7.0) × 10^−2^	14.9	2.7	96–98	[This work]
		Carvacrol	8.9 × 10^−4^	0.9995	(0.3–7.0) × 10^−2^	15.2	2.6	94–101	

^a^ Hydro distillation-headspace solvent microextraction gas chromatography-flame ionization detector. ^b^ Ultrasound-assisted microextraction-nano material solid phase dispersion high-performance liquid chromatography. ^c^ Headspace solid-phase microextraction gas chromatography-mass spectrometry. ^d^ Vortex-assisted surfactant-enhanced dispersive liquid–liquid microextraction high-performance liquid chromatography. ^e^ Nano-sized magnetic molecularly imprinted polymer-dispersive solid phase microextraction-high performance liquid chromatography. ^f^ Ultrasound-assisted dispersive liquid–liquid microextraction by solidifying floating organic droplets gas chromatography-flame ionization detector. ^g^ Retention time. ^h^ Relative standard deviation.

## Data Availability

The original contributions presented in this study are included in the article; further inquiries can be directed to the corresponding author.
